# Supplementary material for: Early warning and proactive control strategies for power blackouts caused by gas network malfunctions
Source: Nat Commun. 2024 Jun 3;15:4714. doi: 10.1038/s41467-024-48964-0 (PMC11148067; doi:10.1038/s41467-024-48964-0)
Supplement: Supplementary file 1 — Supplementary Information [file 41467_2024_48964_MOESM1_ESM.pdf]

Supplementary Information

Early Warning and Proactive Control Strategies for Power Blackouts Caused by Gas Network Malfunctions

Fengshuo Yu[1], Qinglai Guo[1], Jianzhong Wu[2], Zheng Qiao[1], Hongbin Sun[1, 3*]

[1]Department of Electrical Engineering, Tsinghua University, Beijing, China
[2]School of Engineering, Cardiff University, Cardiff, the UK
[3]College of Electrical and Power Engineering, Taiyuan University of Technology, Taiyuan, China
[*]Corresponding author - shb@tsinghua.edu.cn

The rationality of the hypothesis when estimating the early warning indicators in the Methods section can be considered as follows. The basis for this hypothesis is the mass conservation, the momentum conservation and the gas state equations, which together constitute the isothermal model of the gas pipeline commonly used in the industrial field [1]:

$$\begin{cases} \dfrac{\partial \rho}{\partial t} + \dfrac{\partial (\rho v)}{\partial x} = 0 \\ \dfrac{\partial (\rho v)}{\partial t} + \dfrac{\partial (\rho v^2)}{\partial x} + \dfrac{\partial p}{\partial x} + \dfrac{\lambda \rho v |v|}{2D} + \rho g \sin\theta = 0 \\ p = ZRT\rho \end{cases} \tag{1}$$

The independent variable includes time coordinate t and space coordinate x, the dependent variable includes density ρ, flow speed v and pressure p, and the parameters include pipeline friction coefficient λ, inner diameter D, gravity acceleration g, slope angle θ, compression factor Z, gas constant R and temperature T. By substituting variables, we can get the form of the telegraph equations, which is essentially a wave equation with the propagation speed $c = \sqrt{ZRT}$.

The exact expression of our assertion is that in the absence of gas flow within a pipe, encompassing both the gas source and the gas load, the pressure across various points will tend to equalize. In other words, if the terminal segment is only connected to the impacted gas turbine, there is almost no pressure gradient in the segment when the gas turbine shuts down. This phenomenon is better understood considering that the speed of gas pressure wave is approximately the speed of sound (about 300 m/s), which can be proved by expressing the dynamic equations of gas pipelines in the form of a wave equation [2]. Gas pressure waves undergo multiple reflections in the gas pipeline over a short period, leading to a tendency for uniform pressure at each point along the pipeline.

In our examination of the terminal area linked to the gas turbine, the AET, denoting the time for the gas turbine to cease operation due to insufficient pressure, is a key factor in our optimization model. The model reveals that the mass flow of gas turbines is usually reduced steadily in the proactive control strategy. When the gas turbine undergoes shutdown, its impact on the gas flow within the pipeline is typically minimal, validating the plausibility of this hypothesis. As an illustration,

consider the gas pipeline depicted in **Supplementary Figure 1**. Let's assume that the gas supply is lost at the head end, transforming it into a point where the flow boundary condition is zero. Subsequently, the flow rate of the terminal gas turbine decreases at a rate of 5% capacity per minute. **Supplementary Figure 2** depicts the pressure curve for each point along the pipeline at different times, and **Supplementary Table 1** shows the standard deviation of pressure per kilometre. That is to say, after the failure of the gas source, the pressure in the pipeline tends to be uniform.

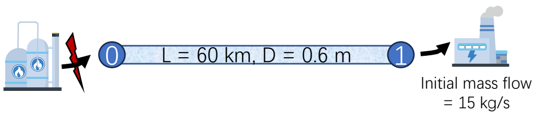

Supplementary Fig. 1 | Parameters of the single-pipe verification case. In this case, there is only one gas turbine at the terminal end of the pipeline whose initial mass flow rate is 15 kg/s, and the flow rate decreases at a rate of 5% capacity per minute.

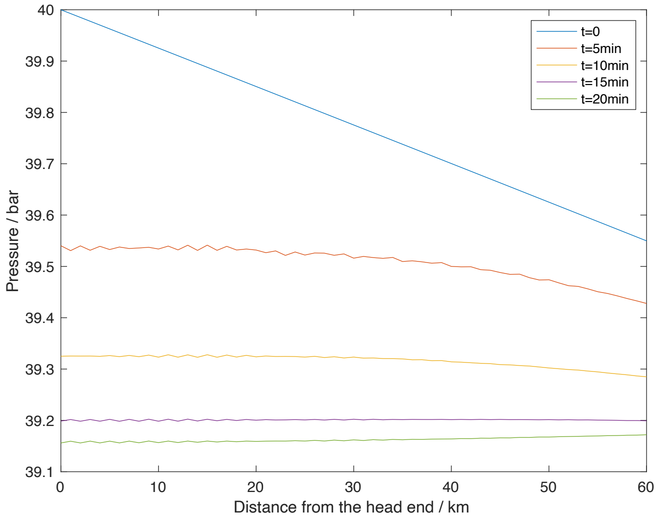

Supplementary Fig. 2 | Pressure curves at different times in the single-pipe case. It can be seen that with the passage of time, the pressure difference at each point in the pipeline shows a decreasing trend. Source data are provided as a Source Data file.

Supplementary Table 1 Standard deviation of pressure per kilometre at different times in the single-pipe case.

Time / min	0	5	10	15	20
Standard deviation / bar	0.1332	0.0321	0.0121	0.0012	0.0045

The situation becomes more intricate when there is a constant gas load in the terminal area. If the shutdown pressure of the constant gas load is lower than that of the gas turbine, gas flow inside the pipeline persists when the gas turbine is shut down. However, there is typically a certain distance between the gas turbine and other gas loads. Consequently, the pressure valley in the terminal area will also be situated at a distance from the gas turbine, generally preventing the pressure at the gas turbine location from dropping below the average pressure across the entire area. This usually results in a larger estimate of the final line pack and a smaller estimate of the available line pack. Despite

leading to a smaller estimate of the AET, which indicates a more 'urgent' failure determination, it still aligns with the requirements of proactive control. As an illustration, consider the gas network depicted in **Supplementary Figure 3**. Let's assume that the gas supply is lost at the head end. Subsequently, the flow rate of the terminal gas turbine decreases at a rate of 5% capacity per minute. **Supplementary Figure 4** depicts the pressure curve for each point in the network at different times, and **Supplementary Table 2** shows the ratio between the inlet pressure of the gas turbine and the average pressure of the whole network. It can be seen that after the gas source failure occurs, the error between the gas turbine inlet pressure and the mean pressure of the network is very small and decay rapidly, so it is feasible to estimate the final line pack with the minimum inlet pressure of the gas turbine.

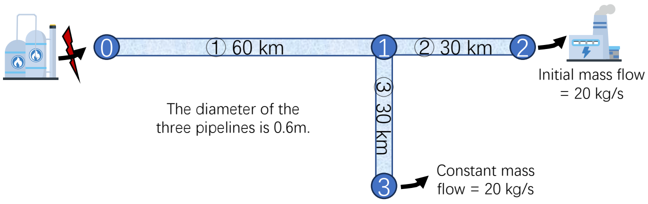

Supplementary Fig. 3 | Parameters of the multi-pipe verification case. In this case, there is both a non-generator constant gas load (20 kg/s) as well as a gas turbine in the network. The mass flow rate of the gas turbine is 20 kg/s at the initial moment, and decreases at a rate of 5% capacity per minute.

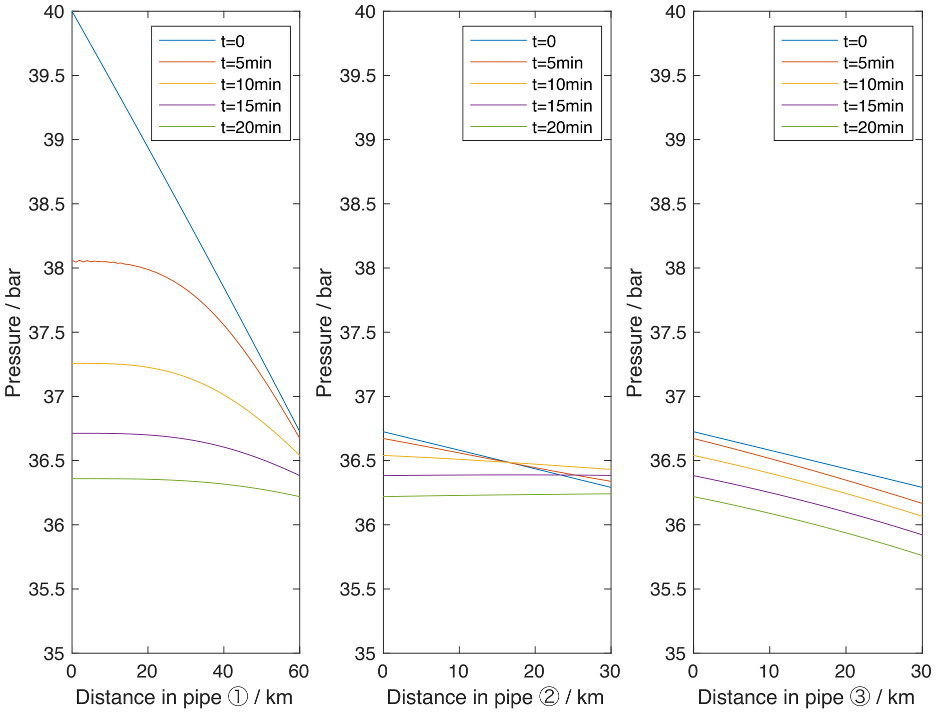

Supplementary Fig. 4 | Pressure curves at different times in the multi-pipe case. It can be seen that for this common scenario in practical engineering, the difference between the inlet pressure of the gas turbine and the average pressure of the whole network is not large even after a short period of time. Source data are provided as a Source Data file.

Supplementary Table 2 Standard deviation of pressure per kilometre at different times in the multi-pipe case.

Time / min	0	5	10	15	20
Pressure ratio / bar	0.9691	0.9805	0.9918	0.9982	1.0006

We also compare the SAET calculated under this hypothesis with the time needed for gas turbine inlet pressure to drop to the threshold through the dynamic simulation method. **Supplementary Figure 5** illustrates the topology of the gas network, and **Supplementary Table 3** gives the calculation results of the SAET for various failures. The gas source pressure is set at 2 MPa. The ordinary gas loads m_{L1}, m_{L2}, and m_{L3} are 5.98, 4.98, and 2.99 kg/s, respectively. The output of each gas turbine is 80 MW with a thermal efficiency of 0.55, and both of them have a minimum inlet pressure of 1.5 MPa. Branch ⑧ is a compressor with an output pressure of 2 MPa and a compression ratio ranging from 1.1 to 2.0. Assuming that the valves at the head end of pipes ①, ⑤, or ⑥ are closed, and the SAET of gas turbines G1 and G2 are calculated by both the approximate method and the hydraulic simulation. It can be seen that the error caused by the approximation is very small.

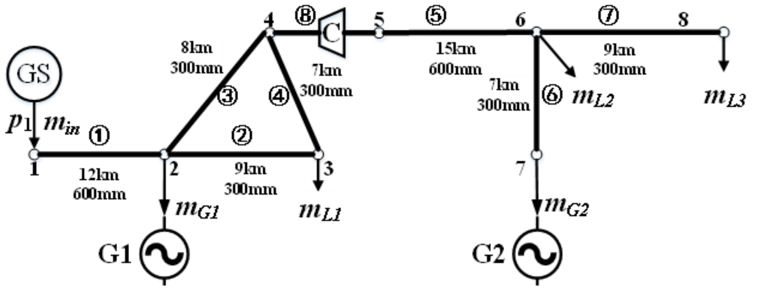

Supplementary Fig. 5 | Topology and parameters of the large verification case. In this case, the valves at the head end of pipes ①, ⑤, or ⑥ are closed, respectively, and the SAET of G1 and G2 are calculated.

Supplementary Table 3 Comparison of the SAET under different pipeline failures.

**Pipeline serial number**			①	⑤	⑥
**SAET (min)**	G1	Approximate calculation method	15.68	/	/
		Dynamic hydraulic simulation	15.10	/	/
	G2	Approximate calculation method	57.63	30.48	11.14
		Dynamic hydraulic simulation	58.57	30.50	11.17

In essence, our introduction of early warning indicators aims to 'condense' the information of the gas network malfunction within an acceptable margin of error. This method provides the proactive control parameters for the power system in a comprehensible manner without significantly compromising the privacy of the gas system. If we want to make the early warning more accurate, it is necessary to introduce a more complete dynamic hydraulic analysis of the gas network, and

update the boundary conditions of coupling points iteratively. For proactive control after the failure, this method is difficult to meet the time requirement.

Reference:

[1] Zhu, G.Y., Henson, M.A., Megan, L., 2001. Dynamic modeling and linear model predictive control of gas pipeline networks. J. Process Control 11, 129-148.

[2] B. Chen et al. Energy-Circuit-Based Integrated Energy Management System: Theory, Implementation, and Application. Proceedings of the IEEE 110, 1897-1926 (2022). https://doi.org/10.1109/JPROC.2022.3216567.
