# Supplementary material for: Early warning and proactive control strategies for power blackouts caused by gas network malfunctions
Source: Nat Commun. 2024 Jun 3;15:4714. doi: 10.1038/s41467-024-48964-0 (PMC11148067; doi:10.1038/s41467-024-48964-0)
Supplement: Supplementary file 3 — Reporting Summary [file 41467_2024_48964_MOESM3_ESM.pdf]

nature portfolio

Corresponding author(s): Hongbin Sun

Last updated by author(s): Apr 25, 2024

Reporting Summary

Nature Portfolio wishes to improve the reproducibility of the work that we publish. This form provides structure for consistency and transparency in reporting. For further information on Nature Portfolio policies, see our Editorial Policies and the Editorial Policy Checklist.

Statistics

For all statistical analyses, confirm that the following items are present in the figure legend, table legend, main text, or Methods section.

n/a	Confirmed	
☒	☐	The exact sample size (*n*) for each experimental group/condition, given as a discrete number and unit of measurement
☒	☐	A statement on whether measurements were taken from distinct samples or whether the same sample was measured repeatedly
☒	☐	The statistical test(s) used AND whether they are one- or two-sided<br>*Only common tests should be described solely by name; describe more complex techniques in the Methods section.*
☒	☐	A description of all covariates tested
☒	☐	A description of any assumptions or corrections, such as tests of normality and adjustment for multiple comparisons
☒	☐	A full description of the statistical parameters including central tendency (e.g. means) or other basic estimates (e.g. regression coefficient) AND variation (e.g. standard deviation) or associated estimates of uncertainty (e.g. confidence intervals)
☒	☐	For null hypothesis testing, the test statistic (e.g. *F*, *t*, *r*) with confidence intervals, effect sizes, degrees of freedom and *P* value noted<br>*Give P values as exact values whenever suitable.*
☒	☐	For Bayesian analysis, information on the choice of priors and Markov chain Monte Carlo settings
☒	☐	For hierarchical and complex designs, identification of the appropriate level for tests and full reporting of outcomes
☒	☐	Estimates of effect sizes (e.g. Cohen's *d*, Pearson's *r*), indicating how they were calculated

Our web collection on statistics for biologists contains articles on many of the points above.

Software and code

Policy information about availability of computer code

Data collection	No software was used.
Data analysis	MATLAB 2023b

For manuscripts utilizing custom algorithms or software that are central to the research but not yet described in published literature, software must be made available to editors and reviewers. We strongly encourage code deposition in a community repository (e.g. GitHub). See the Nature Portfolio guidelines for submitting code & software for further information.

Data

Policy information about availability of data

All manuscripts must include a data availability statement. This statement should provide the following information, where applicable:

- Accession codes, unique identifiers, or web links for publicly available datasets
- A description of any restrictions on data availability
- For clinical datasets or third party data, please ensure that the statement adheres to our policy

The input dataset is available for download at https://github.com/YuFengshuo/gas_electric_early_warning, including statistics of global gas infrastructure and topology and parameters of the coupled gas-electricity system in Zhejiang Province.

Research involving human participants, their data, or biological material

Policy information about studies with human participants or human data. See also policy information about sex, gender (identity/presentation), and sexual orientation and race, ethnicity and racism.

Reporting on sex and gender	This information has not been collected.
Reporting on race, ethnicity, or other socially relevant groupings	This information has not been collected.
Population characteristics	This information has not been collected.
Recruitment	This information has not been collected.
Ethics oversight	This information has not been collected.

Note that full information on the approval of the study protocol must also be provided in the manuscript.

Field-specific reporting

Please select the one below that is the best fit for your research. If you are not sure, read the appropriate sections before making your selection.

☐ Life sciences ☐ Behavioural & social sciences ☒ Ecological, evolutionary & environmental sciences

For a reference copy of the document with all sections, see nature.com/documents/nr-reporting-summary-flat.pdf

Ecological, evolutionary & environmental sciences study design

All studies must disclose on these points even when the disclosure is negative.

Study description	During a gas-electric cascading failure, the gas stored in pipelines becomes a buffer for pressure variations, which typically makes the time required for the propagation and escalation of the malfunction much longer than the time needed for the electric power control centre (EPCC) to acquire failure information from the gas system. In contrast, the power system can swiftly adapt its operational state, offering the potential to proactively mitigate the gas-electric cascading failure. These facts inspired us to develop the gas-electric early warning system described in this paper. Compared to conventional security control methods, the gas-electric early warning system makes critical use of the time delay between the gas network malfunction and its impact on gas turbines. Once the early warning system is introduced, the EPCC can employ proactive control before the failure reaches the power system, rather than passive control after the electric failure occurs. In the former scenario, the impacts of gas network malfunctions on the electric power system will be significantly reduced relative to the scenario without the early warning system.
Research sample	The study in this paper does not address this.
Sampling strategy	The study in this paper does not address this.
Data collection	The study in this paper does not address this.
Timing and spatial scale	The study in this paper does not address this.
Data exclusions	The study in this paper does not address this.
Reproducibility	The study in this paper does not address this.
Randomization	The study in this paper does not address this.
Blinding	The study in this paper does not address this.

Did the study involve field work? ☐ Yes ☒ No

Reporting for specific materials, systems and methods

We require information from authors about some types of materials, experimental systems and methods used in many studies. Here, indicate whether each material, system or method listed is relevant to your study. If you are not sure if a list item applies to your research, read the appropriate section before selecting a response.

Materials & experimental systems

n/a	Involved in the study
☒	☐ Antibodies
☒	☐ Eukaryotic cell lines
☒	☐ Palaeontology and archaeology
☒	☐ Animals and other organisms
☒	☐ Clinical data
☒	☐ Dual use research of concern
☒	☐ Plants

Methods

n/a	Involved in the study
☒	☐ ChIP-seq
☒	☐ Flow cytometry
☒	☐ MRI-based neuroimaging
